# Musculoskeletal Assessment in Patients with Adrenal Incidentalomas: Should We Integrate the Trabecular Bone Score and/or Circulating Irisin?

**DOI:** 10.3390/diagnostics16050761

**Published:** 2026-03-03

**Authors:** Alexandra-Ioana Trandafir, Oana-Claudia Sima, Dana Manda, Mihai Costachescu, Veronica Cumpata, Ana Valea, Sorina Violeta Schipor, Claudiu Nistor, Ana Popescu, Emi Marinela Preda, Mara Carsote

**Affiliations:** 1 PhD Doctoral School of “Carol Davila” University of Medicine and Pharmacy, 020021 Bucharest, Romania; alexandra-ioana.trandafir@drd.umfcd.ro; 2 Department of Clinical Endocrinology V, “C.I. Parhon” National Institute of Endocrinology, 011863 Bucharest, Romania; oana-claudia.sima@drd.umfcd.ro; 3 Department of Research, “C.I. Parhon” National Institute of Endocrinology, 011863 Bucharest, Romania; dana.manda@parhon.ro (D.M.); sorina.schipor@parhon.ro (S.V.S.); 4 Department of Radiology and Medical Imaging, “Dr. Carol Davila” Central Military University Emergency Hospital, 010825 Bucharest, Romania; mihai.costachescu@drd.umfcd.ro; 5 Department of Family Medicine, State “Nicolae Testemiţanu” University of Medicine and Pharmacy, 2004 Chisinau, Moldova; veronica.cumpataciorba@gmail.com (V.C.); ana.popescu@usmf.md (A.P.); 6 Department of Endocrinology, “Iuliu Hatieganu” University of Medicine and Pharmacy, 400012 Cluj-Napoca, Romania; ana.valea@umfcluj.ro; 7 Department of Endocrinology, County Emergency Clinical Hospital, 400347 Cluj-Napoca, Romania; 8 Department 4-Cardio-Thoracic Pathology, Thoracic Surgery II Discipline, “Carol Davila” University of Medicine and Pharmacy, 050474 Bucharest, Romania; claudiu.nistor@umfcd.ro; 9 Thoracic Surgery Department, “Dr. Carol Davila” Central Military University Emergency Hospital, 010242 Bucharest, Romania; 10 Department of Radiology, “Carol Davila” University of Medicine and Pharmacy, 050474 Bucharest, Romania; 11 Department of Radiology and Medical Imaging, “Foisor” Clinical Hospital of Orthopaedics, Traumatology and Osteoarticular TB, 021382 Bucharest, Romania; 12 Department of Endocrinology, “Carol Davila” University of Medicine and Pharmacy, 020021 Bucharest, Romania

**Keywords:** bone, osteoporosis, TBS, DXA, myokine, exerkine, biomarker, cortisol, ACTH, adrenal tumor

## Abstract

**Background/Objectives**: Current musculoskeletal health assessment expanded beyond bone mineral density (BMD) at central DXA to include, for instance, trabecular bone score (TBS) and emergent biomarkers, such as adipokines and myokines (e.g., irisin) assays. A current gap in their application is reflected in limited research regarding adrenal tumors, especially non-functional adrenal tumors/mild autonomous cortisol secretion (NFATs/MACS). To assess this current gap, we aimed to explore beyond BMD, specifically, TBS and circulating irisin, in relation to the adrenal status in NFATs/MACS. **Methods**: This is a prospective, cross-sectional, single-center, exploratory study, conducted between October 2024 and December 2025. **Results**: A total of 81 menopausal women were included (mean age of 63.26 ± 8.82 years, 15.86 ± 9.5 years since menopause, average BMI of 30.69 ± 5.76 kg/sqcm. Out of them, 33.33% had NFATs/MCAS (group AI) and 66.67% were controls (group C), with similar age, years since menopause, and BMI. The prevalence of type 2 diabetes was 66.67% versus 68.52% (*p* = 0.865). TBS correlated with lumbar BMD/T-score (*N* = 33), while age and lumbar BMD were independent TBS predictors (*N* = 81), but not type 2 diabetes nor NFAs/MCAS. TBS correlated with the five-year age groups (r = −0.273, *p* = 0.003). Irisin correlated with osteocalcin (r = −0.252, *p* = 0.007), P1NP (r = −0.187, *p* = 0.049) and CrossLaps (r = −0.209, *p* = 0.026) in tumor-free controls. In the AI group, a higher irisin was associated with a higher second-day cortisol after 1 mg DST (r = 0.11, *p* = 0.584) and a lower ACTH (r = −0.716, *p* < 0.001). The rate of low TBS (based on 1.350 cutoffs) was 48.15% versus 38.89% in group AI versus C. In the AI group, patients with low TBS had lower osteocalcin, P1NP, and CrossLaps than those with normal TBS, with a similar rate of type 2 diabetes (which might reduce the bone turnover markers) and MACS-positive prevalence (between 25 and 28%). **Conclusions**: The median glycated hemoglobin A1c (5.78% versus 5.93%, *p* = 0.94) and median HOMA-IR (1.53 versus 1.42, *p* = 0.948) suggest a certain level of glucose control, which might not be reflected in severely damaged bone microarchitecture, as shown by TBS. Irisin may be one of the additional factors in these tumors reflecting the hormonal burden. Irisin was statistically significantly elevated with the increase in BMI groups. To our best awareness, this is the first synchronous analysis of TBS and irisin levels in this type of tumor to address the bone status in relation to the glucose profile and adrenal panel. Noting this is an exploratory, hypothesis-generating study, further research will highlight the true value of TBS and irisin for practitioners in the adrenal field, including multi-layered models of bone status prediction.

## 1. Introduction

Modern approach of the musculoskeletal health assessment in daily practice expanded beyond bone mineral density (BMD) at central Dual-Energy X-Ray Absorptiometry (DXA), for instance, to the lumbar DXA-derived trabecular bone score (TBS) or hip geometry, and to emergent biomarkers, others than the traditional bone turnover markers, such as adipokines and exerkines (myokines) assays [1,2,3]. They might contribute to the bone formation/resorption process, either directly or indirectly via crossroads with glucose metabolism, chronic inflammation, and oxidative stress [4,5,6,7]. Among this panel, irisin is a novel muscle-released hormone, which has been placed in relationship with multiple metabolic pathways under physiological and pathological circumstances (e.g., physical exercise/training or cold exposure, respectively, sarcopenia, diabetes, osteoporosis, obesity, cancers, etc.); however, its testing is not yet standardized [8,9,10,11].

On the other hand, an increasing number of scans amid various imaging tools has led to a rising rate of incidentalomas; for instance, accidentally detected adrenal masses are reported in 1–5% to 10% of the abdominal scans. More than half of this neoplasia involves non-secretor benign tumors of the adrenal cortex (true adrenal incidentalomas from both an imaging and a hormonal perspective). Further on, approximately one third of these non-functionally adrenal tumors might actually show a certain level of hormonal activity, designed as mild autonomous cortisol secretion (MACS), which was previously named subclinical Cushing’s syndrome. Notably, the non-functioning pattern according to the dexamethasone (DST) suppression test might be sub-optimally characterized due to a heterogeneous spectrum of tumor-related secretion and imperfect diagnosis tools we currently have [12,13,14].

The landscape of comorbidities in these adrenal incidentalomas varies from a higher cardio-metabolic risk to an increased risk of osteoporosis and osteoporotic fractures, which is more prevalent in certain population subgroups (e.g., menopausal women), noting that, generally, the bone status is less understood [15,16,17]. The diagnosis of osteoporosis in such patients does not represent an indication of adrenalectomy, nor has a preferred interventional strategy in MACS-related osteoporosis been promoted at the level of guideline-based management [18,19,20,21]. To date, only a limited number of studies approached the topic of non-BMD assessment, particularly TBS, in individuals with adrenal incidentalomas [22,23,24,25,26,27], and even fewer studied the blood/circulating irisin profile in these subjects [28].

### Objective

To assess the current gap of the musculoskeletal health evaluation beyond BMD, we aimed to explore TBS and circulating irisin in relation to the adrenal status in patients diagnosed with adrenal incidentalomas.

## 2. Materials and Methods

### 2.1. Study Design

This is a prospective, cross-sectional, single-center, clinical, exploratory, and pilot study, conducted between October 2024 and December 2025.

### 2.2. Study Population

Menopausal women were enrolled based on the following inclusion criteria: (1) accidental detection of an adrenal tumor at abdominal computed tomography (CT); (2) confirmation of a non-functional adrenal tumor based on plasma morning cortisol level after 1 mg dexamethasone (DST) inhibition test less than 5 µg/dL; (3) an age of 50 years or older; and (4) the provision of informed written consent for the study enrollment.

Exclusion criteria were as follows: (1) active or prior malignancies, inflammatory, autoimmune diseases, type 1 or secondary diabetes, secretor endocrine tumors (including Cushing’s syndrome or Cushing’s disease); (2) prior bariatric surgery; (3) previous or current medication against diabetes and/or obesity, and glucocorticoid exposure; (4) unilateral or bilateral adrenalectomy or the co-presence of bilateral adrenal tumor masses; (5) prior or synchronous diagnosis of osteoporosis/osteoporotic fracture, exposure to medication against osteoporosis; (6) suspected or confirmed adrenal malignancy (a tumor diameter larger than 4 cm, the presence of abdominal lymph nodes involvement, high attenuation, slow washout after contrast, irregular borders, local invasion, tumor necrosis/hemorrhage, irregular borders) or adrenal cyst at abdominal CT scan; (7) bone or renal metabolic diseases; and (8) non-interpretable central DXA scan.

The entire cohort included the study group with adrenal incidentalomas (group AI) and controls [subjects who did not present any adrenal tumor during a standardized (intravenous-contrast) abdominal CT scan and fulfilled all the other inclusion/exclusion criteria].

### 2.3. Study Protocol

**Figure 1:**
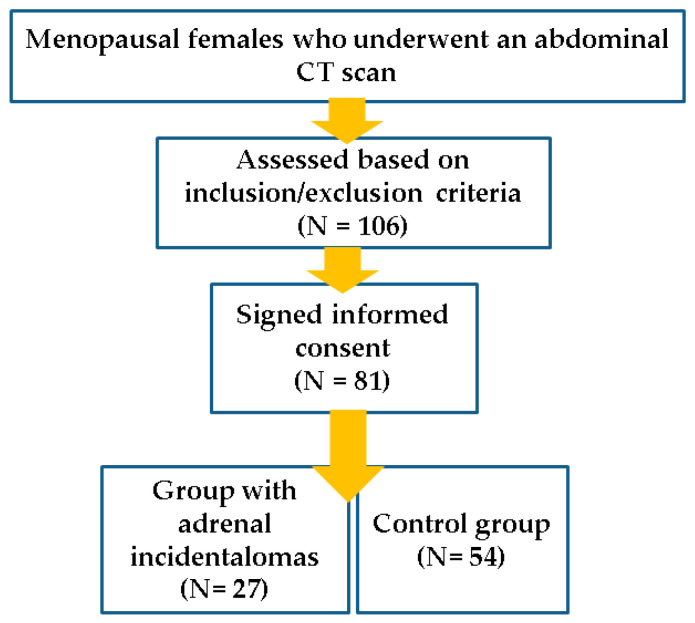
Schematic representation of the study protocol.

Menopausal females who underwent an abdominal CT scan (*N* = 260) were evaluated based on the mentioned inclusion/exclusion criteria (*N* = 106). After specific informed consent was signed, the blood tests were performed: fasting assays (after 8–12 h of fasting), as well as a 75 g oral glucose tolerance test (OGTT) in subjects who were not previously known with diabetes or had a fasting glycaemia of less than 125 mg/dL (*N* = 81) (Figure 1).

**Data collection**: Information was collected on age (years), menopause duration (years since menopause), and body mass index (BMI) calculation [height/(weight)^2^ in kg/sqm]. We applied the standard BMI (kg/sqm) categories: normal (BMI ≥ 18.5 ≤ 24.9), overweight (BMI ≥ 25.0 ≤ 29.9), grade I obesity (BMI ≥ 30.0 ≤ 34.9), grade II obesity (≥35.0 ≤ 39.9), and grade III obesity (BMI ≥ 40.0) [29].

**Glucose profile**: Measurements included glycated hemoglobin (%) (turbidimetry, Roche); glycaemia (photometry, Roche); and insulin [chemiluminescence immunoassay (CLIA), Beckman Coulter]. The latter two were tested fasting and at 60 min and 120 min during a 75 g oral glucose tolerance test. The calculation of homeostasis model assessment of insulin resistance (HOMA-IR) was based on the formula: fasting glycaemia (mg/dL) X fasting insulin (µI/mL)/405. Additional biochemistry panel included fasting total proteins, urea, and creatinine [all based on the photometry method (Abbott]. The subjects with a 2 h glycaemia during the oral glucose tolerance test of 200 mg/dL or above were confirmed with type 2 diabetes [30].

**Adrenal profile** included morning plasma ACTH and cortisol, and second-day plasma cortisol after 1 mg DST [both hormone tests were based on electrochemiluminescence immunoassay (ECLIA), Roche]. MACS were considered based on a value of second-day cortisol after 1 mg DST between ≥1.8 µg/dL and <5 µg/dL [31]. CT scan provided the largest tumor diameter (mm).

**Musculoskeletal profile:** Mineral metabolism assays included total and ionic serum calcium (photometry, Roche), serum phosphorus (photometry, Abbott), 25-hydroxyvitamin D (CLIA, DiaSorin), and parathormone (ECLIA, Roche). Blood bone turnover makers included the markers of formation [total alkaline phosphatase (photometry, ROCHE), osteocalcin (ECLIA, Roche), P1NP (procollagen 1 N-terminal propeptide) (ECLIA, Roche)] and resorption (CrossLaps) (ECLIA, Roche).

**Irisin testing** [enzyme-linked immunosorbent assay (ELISA), MyBioSource, Inc., San Diego, CA 92195-3308, USA, MBS706887] presented the detection range of 3.12 ng/mL to 200 ng/mL and required a 1:5 dilution for a blood level of >200 ng/mL (intra- and inter-assay precision of <8%, respectively, of <10%).

**Central DXA** was performed (GE Lunar Prodigy device) at L1–L4 lumbar spine, total hip, and femoral neck, and provided BMD and T-score. Osteoporosis/osteopenia was defined according to current standard guidelines (a T-score of −2.5 or below, or a T-score between −1 and −2.5, respectively) [32]. TBS was generated by DXA machine-attached software, TBS iNsight (ver3). “Low” TBS was used for a value of 1.350 or below (group LT), and “normal” TBS was based on a value higher than 1.350 (group NT) [33]. Vertebral fractures screening was based on a profile thoracic–lumbar spine X-Ray. Central DXA scans, spine X-Rays, and adrenal CT scans were double checked by two trained radiologists (M.K. and E.M.P.).

### 2.4. Statistical Aspects

Continuous variables were summarized either as mean with standard deviation (SD) or as median with quartiles (Q1, Q3), depending on data distribution, while categorical variables were reported as frequencies and percentages. Associations between categorical variables were evaluated using the chi-square test or Fisher’s exact test, depending on expected cell counts. For comparisons between two independent groups, either Student’s *t*-test or the Mann–Whitney U test was applied, as appropriate. Differences across multiple groups were assessed using one-way analysis of variance (ANOVA) or the Kruskal–Wallis test. Relationships among numerical variables were explored using Kendall’s tau correlation coefficient. Multivariable associations were investigated through multiple linear regression analyses, with results reported as unstandardized regression coefficients (B) with corresponding standard errors (SE), alongside standardized beta coefficients (β) to facilitate comparison of effect sizes. Model performance was assessed using the coefficient of determination (R-squared), reflecting the proportion of variance explained by the independent variables. All statistical analyses were conducted using GraphPad Prism version 10.6.0 (GraphPad Software, Boston, MA, USA), SPSS version 29.0.2.0 (IBM, Armonk, NY, USA), and Microsoft Excel version 16.104 (Microsoft, Redmond, WA, USA). Statistical significance was defined by a two-sided *p*-value below 0.05.

### 2.5. Ethical Approval

The Ethical Committee of “C.I. Parhon” National Institute of Endocrinology, Bucharest, approved the study (approval number 32, dated 30 September 2024).

## 3. Results

A total of 81 patients were included in the final analysis, with a mean age of 63.26 ± 8.82 years, 15.86 ± 9.50 years since menopause, and a mean BMI of 30.69 ± 5.76 kg/sqcm. About 33.33% of the patients had an adrenal incidentaloma (group AI; age range: 50 to 79 years), and 66.67% were controls (group C; age range: 50 to 77 years), with similar age, years since menopause, and BMI between the groups (Table 1).

**Figure 2:**
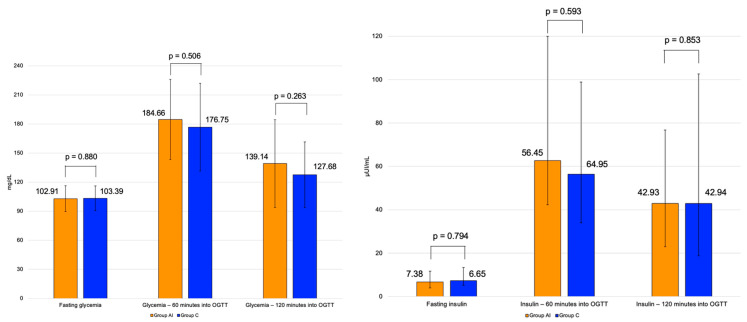
Bar charts with error bars showing mean and standard deviations of fasting and oral glucose tolerance test—the levels of glycaemia (**left**) and insulin (**right**) in group AI (orange) versus group C (blue).

Mean glycated hemoglobin was similar in group AI of 5.78 ± 0.29% and group C of 5.93 ± 0.28%. 66. There was no statistically significant between-group difference for glycemia and insulin at 60 min and 120 min in OGTT (Figure 2).

The adrenal profile in the AI group showed an average tumor diameter of 2.3 cm (Table 2).

Circulating irisin had a median value of 71.17 (20.16, 167.86) ng/mL in group AI and was similar to 77.64 (34.92, 133.28) ng/mL in group C. No between-group difference was found with respect to the mineral metabolism assays and bone turnover markers (Table 3).

Mean TBS value was similar in group AI of 1.342 ± 0.088 versus group C of 1.362 ± 0.096. The rate of osteoporosis at DXA was of 12.35% (*N* = 81), but the mean T-score at each central DXA site was found in the normal DXA category for each group AI and C (Table 4).

### 3.1. Simple and Multiple Regression Analysis

Within group AI, irisin showed a statistically significant negative correlation with baseline ACTH (r = −0.438, *p* = 0.041) and a positive correlation with second-day plasma cortisol after 1 mg DST (r = 0.716, *p* < 0.001). TBS was statistically significantly and positively correlated with CrossLaps (r = 0.364, *p* = 0.018), lumbar BMD (r = 0.451, *p* = 0.003), and lumbar T-score (r = 0.420, *p* = 0.005).

Within group C, irisin was statistically significantly negatively correlated with osteocalcin (r = −0.252, *p* = 0.007), P1NP (r = −0.187, *p* = 0.049), and CrossLaps (r = −0.209, *p* = 0.026) (Table 5).

The multiple linear regression analysis for identifying independent predictors of TBS explained 23.6% of the variance in TBS and was statistically significant (R-squared = 0.236, *p* = 0.003). An increase in age by 1 year was statistically significantly and independently associated with a decrease in TBS of −0.004 ± 0.001 (*p* = 0.003). Lumbar BMD was a statistically significant independent predictor for TBS, showing that an increase of 1 g/sqcm led to an increase in TBS of 0.172 ± 0.070 (*p* = 0.017). Type 2 diabetes or adrenal incidentaloma were not statistically significant predictors in the model. Age was the most important predictor in the model, with the highest β of −0.358 (Table 6).

### 3.2. Age-Groups Analysis

Age-groups sub-analysis showed a similar distribution of patients in each group (Table 7).

**Figure 3:**
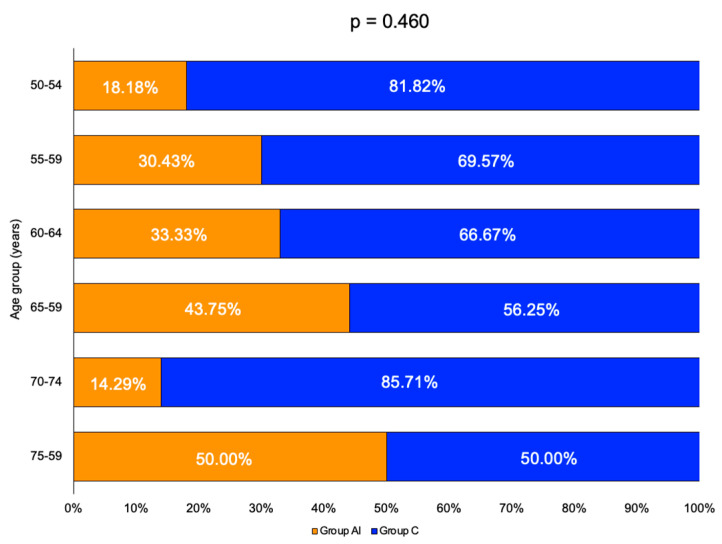
Stacked bar chart showing the percentage of patients from group AI (orange) and group C (blue) in each age group.

The highest rate was found in the 75–79 year age group, with a prevalence of 50% within the AI group (Figure 3).

Irisin and TBS were similar among these age groups (Table 8).

### 3.3. Analysis of Body Mass Index-Based Groups

Sub-analysis of BMI groups showed similar distributions of patients in group AI and group C (Table 9).

Irisin was statistically significantly elevated with the increase in BMI groups (*p* = 0.022) (Table 10).

Irisin was statistically significantly and positively correlated with the BMI groups (r = 0.259, *p* = 0.002) (Table 11).

**Figure 4:**
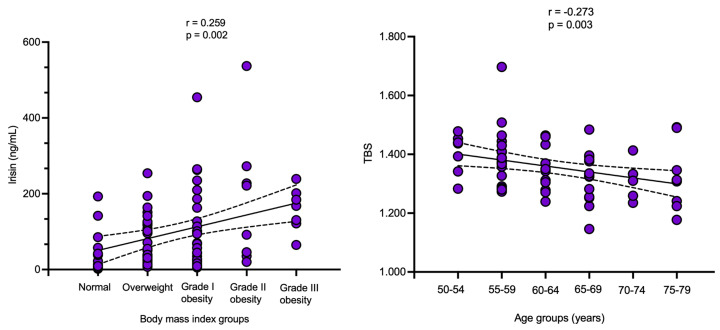
Scatterplot showing the correlation between body mass index groups and irisin (**left**) and TBS (**right**).

TBS showed a statistically significant negative correlation with age groups (r = −0.273, *p* = 0.003) (Figure 4).

### 3.4. Analysis of the Subgroups with Low Versus Normal Trabecular Bone Score

A total of 48.15% of patients from group AI had low TBS (group LT), and 51.85% had normal TBS (group NT). From group C, 38.89% were within group LT, and 61.11% were within group NT. Group LT-C had statistically significantly higher age and more years since menopause versus group NT-C (*p* = 0.003, respectively, *p* = 0.031). Median circulating irisin was similar in LT versus NT, regardless of AI or control group (Table 12).

**Figure 5:**
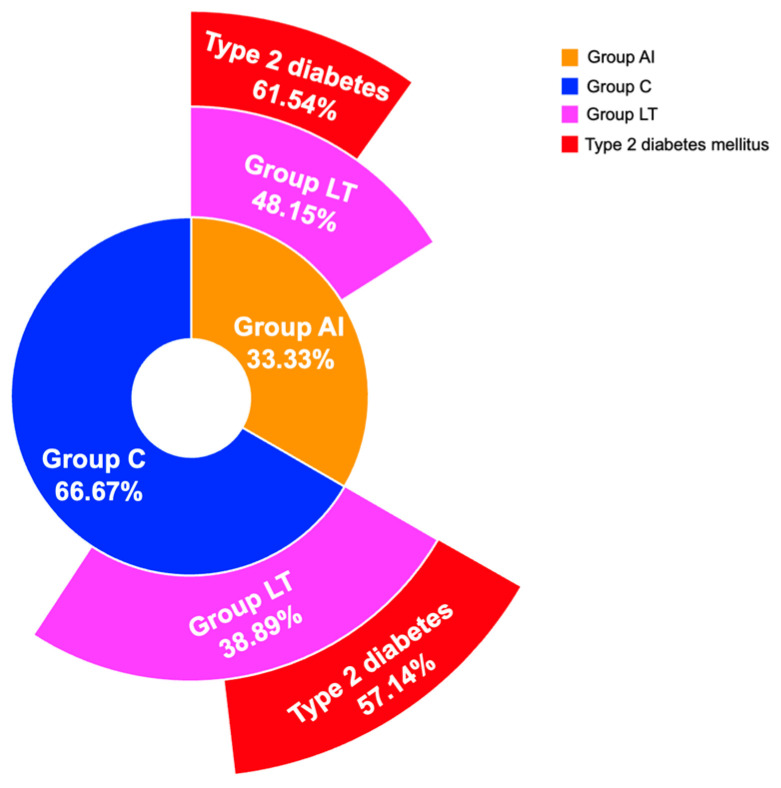
Multi-level donut chart showing type 2 diabetes prevalence in sub-group LT from group AI versus group C.

Glycemic profile, including the rate of type 2 diabetes, was similar between group LT and group NT, in both group AI and group C (Figure 5).

**Figure 6:**
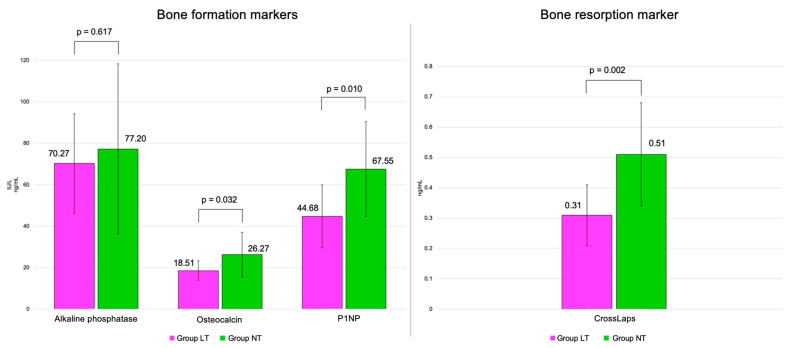
Bar charts with error bars showing mean and standard deviations of bone formation markers and bone resorption markers in patients with low TBS (LT—magenta) versus normal TBS (NT—green) within group AI.

Mean osteocalcin was statistically significantly lower in group LT of 18.51 ± 4.74 ng/mL compared to group NT of 26.27 ± 10.78 ng/mL from group AI (*p* = 0.032), as well as P1NP (44.68 ± 15.21 ng/mL versus 67.55 ± 22.86 ng/mL, *p* = 0.010) and CrossLaps (0.31 ± 0.10 ng/mL versus 0.51 ± 0.17 ng/mL, *p* = 0.002) (Figure 6).

**Figure 7:**
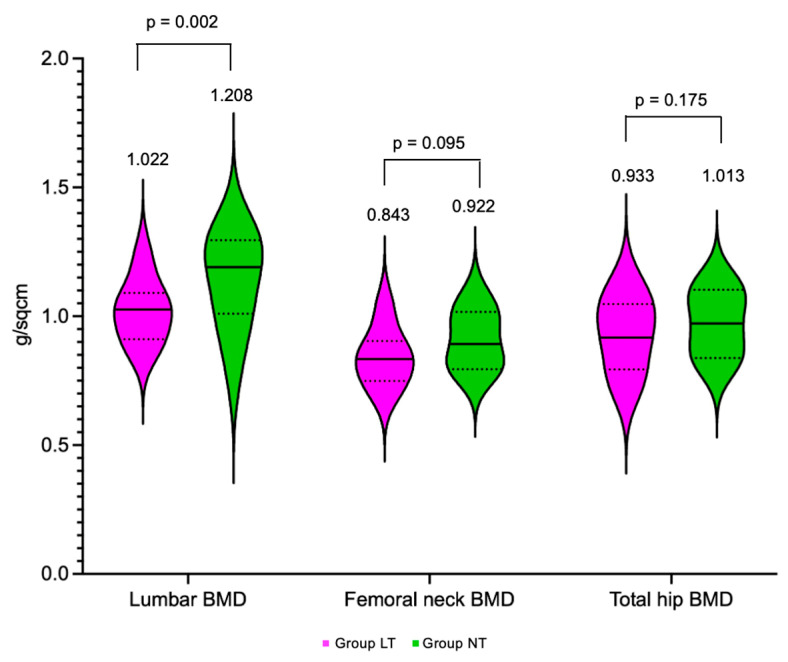
Box violin plots showing lumbar BMD, femoral neck BMD, and total hip BMD in patients with low TBS (LT—magenta) versus normal TBS (NT—green) within group AI.

Within group AI, lumbar BMD (1.022 ± 0.121 g/sqcm) and lumbar T-score (−1.29 ± 0.97 SD) were statistically significantly lower in group LT compared to group NT (1.208 ± 0.123 g/sqcm and 0.10 ± 0.99 SD, respectively; *p* = 0.002, *p* = 0.003). Femoral neck and total hip BMD and T-score were similar between the TBS groups, regardless of group AI or group C (Figure 7).

No between-group difference was registered with regard to the adrenal panel in LT-AI versus NT-AI (Table 13).

## 4. Discussion

In this exploratory analysis of cross-disciplinary interest (adrenal–bone/muscle), we evaluated the role of TBS and circulating irisin as potential clinical markers in subjects confirmed with apparently non-functional adrenal tumors (with a 25% prevalence of MACS-positive profile) versus age- and BMI-matched controls. Overall, 81 individuals were explored (33.33% with a non-secretor adrenal tumor; mean age in the sixth decade, average BMI in the category of grade I obesity, 12% general rate of fracture-free osteoporosis). No between-group difference (tumor-positive versus tumor-free individuals) with respect to the glucose and bone profile, according to the baseline study protocol, as well as circulating irisin level (ELISA) was identified.

Currently, BMD/T-score at DXA remains the gold standard for osteoporosis diagnosis, while TBS, a gray-level texture indicator, displays a guideline-based recommendation in menopausal women with type 2 diabetes [33,34,35,36,37,38]. There is no standard guideline to pinpoint the particular TBS application in individuals confirmed with this type of tumors, noting they are actually Cushing’s syndrome-free and do not display the full picture of endogenous hypercortisolism [39,40,41]. In this particular cohort, TBS may be potentially connected with both the mild hormonal (cortisol) impairment and glucose profile anomalies. Interestingly, we studied a population with a relative high rate of type 2 diabetes (66.67% versus 68.52%, *p* = 0.865), either as a prior diagnosis (yet, the patients were not treated with anti-diabetics) or as a novel diagnosis according to the glycaemia level during oral glucose tolerance test (higher than 200 mg/dL at two hours). However, the median values of glycated hemoglobin A1c (5.78% versus 5.93%, *p* = 0.94) were high–normal, while median HOMA-IR (1.53 versus 1.42, *p* = 0.948) was not consistent with insulin resistance (>2). These findings suggest a certain level of glucose control, which might not be reflected in a severely damaged bone microarchitecture, as shown by TBS. Additionally, TBS correlated with lumbar BMD/T-score (*N* = 27), while age and lumbar BMD were independent TBS predictors (*N* = 81), but not the co-presence of type 2 diabetes nor the adrenal tumor. TBS showed a statistically significant and negative correlation with age groups (r = −0.273, *p* = 0.003). The age-dependent pattern of TBS has been previously described in general, type 2 diabetic, and non-diabetic populations [42].

We identified a limited number of studies regarding TBS use in adrenal incidentalomas [23,24,25,26,27]. For instance, a previous study in 102 patients with adrenal tumors (34 of them were MACS-positive, as defined by different criteria than those we currently use), found that TBS and lumbar BMD were lower in the patients with mild hypercortisolemia versus controls, while TBS negatively correlated with the second-day plasma cortisol after 1 mg DST (*p* = 0.006), irrespectively of BMD at DXA, age, BMI [23], an association which we could not confirm in the study population. Also, Kim et al. [25] showed this inverse correlation in both males and females after adjustment for confounders [25]. Another study in 110 patients with overt Cushing’s syndrome (*N* = 53) versus MACS-positive tumors (which were also defined based on other criteria than those we currently apply) showed a lower rate of reduced TBS in mild versus overt hypercortisolism (*p* = 0.04). Notably, TBS rather than BMD followed the severity of cortisol excess [24], which suggested that the skeletal health assessment in people with adrenal tumors should go beyond BMD, as shown in the current analysis.

The rate of low TBS (TBS ≤ 1.350) was of 48.15% versus 38.89% in group AI versus C. LT group had an increased age and menopause duration versus NT group in patients without adrenal tumors, but not in group AI, which reveals a more complex influence on TBS values in subjects with adrenal incidentalomas. Moreover, in the tumor group, patients with low TBS had lower osteocalcin, P1NP, and CrossLaps than those with normal TBS, which shows that reduced bone turnover is consistent with a lower TBS. Of note, these subgroups had a similar rate of type 2 diabetes (which might reduce the bone turnover markers) [43] and MACS-positive prevalence (between 25% and 28%).

As opposite to TBS, circulating irisin represents a decade-old hormone currently under continuous research, which is not placed in any guideline so far, and only a limited level of statistical evidence points out its relationship with the menopausal bone profile [44,45,46]. Hypothetically, as shown in murine and human experiments, irisin might be connected to the glucose profile anomalies and indirectly to the bone status by pro-bone-forming actions [47,48,49,50,51,52,53]. Whether irisin anomalies accompany the burden of cortisol excess is still an open issue, similar to recent findings for the inflammatory markers that link the steroid profile to the bone status in MACS [54].

According to the present study, while irisin negatively correlated with osteocalcin (r = −0.252, *p* = 0.007), P1NP (r = −0.187, *p* = 0.049), and CrossLaps (r = −0.209, *p* = 0.026) in tumor-free controls, this association was not found in the group with adrenal incidentalomas. However, in this group, higher irisin was associated with higher second-day cortisol levels after 1 mg DST (r = 0.11, *p* = 0.584) and lower baseline ACTH levels (r = −0.716, *p* < 0.001). This suggests that irisin might be one of the additional factors in these tumors reflecting the hormonal burden. Irisin was statistically significantly elevated with the increase in BMI groups (*p* = 0.022).

To the best of our knowledge, only one study explored circulating irisin levels in people with adrenal incidentalomas [28], and no analysis reported irisin in relation to the bone panel in these patients. This was a cross-sectional analysis of irisin and nesfatin-1 in 59 patients with non-functioning adrenal incidentalomas versus 59 age-, gender- and BMI-matched controls. Irisin was higher in the group with tumors versus controls (17.58 ± 4.38 versus 14.04 ± 4.03 pg/mL, *p* < 0.001), and nesfatin was lower (*p* < 0.001). Positive correlations between irisin and carotid intima–media thickness and epicardial adipose tissue were also observed. Notably, irisin was reported in pg/mL using a different ELISA kit (catalog number: SEN576Hu, Cloud-Clone Corp, USCN Life Science, Wuhan, China) [28]. The selection of an adequate irisin kit remains controversial, noting the paucity of clinical studies conducted to date.

### Limitations and Further Research

The sample size and inclusion of menopausal adults might limit the data interpretation amid this pilot (exploratory), single-center study, and future expansion is mandatory. Currently, TBS is mostly applicable after the age of 40 years. Noting that the study population had a high prevalence of type 2 diabetes and a relatively controlled diabetic disease, the bone damage, as reflected by the spine microarchitecture, may be low at this point; thus, a larger diabetic population with adrenal tumors potentially associates wider spectrum of TBS disturbances.

Variations in the biochemical and hormonal assays with the kit, methods, manufacturer, and biological parameters (hydration, sleep pattern, basal level of physical training, circadian rhythm) might introduce a bias in these results. Of important note, there is no unanimous agreement with respect to the best ELISA kit for irisin testing in humans at this point. Whether the anomalies of the cortisol status impair the interpretation of the irisin assay remains an open matter. Also, irisin seemed related to the BMI spectrum; however, we proposed the hypothesis that a more refined irisin analysis regarding the body mass analysis (e.g., lean mass, fat mass) is needed, which can be easily achieved by DXA. Another hypothesis involves irisin and irisin receptors alterations due to mild or severe cortisol secretion anomalies, which remains an area that is poorly understood at the present time.

From a clinical perspective, we excluded patients with prevalent or incident osteoporotic fractures in order to avoid the bias of a complicated osteoporosis; thus, the generalization for the population with fragility fractures might not be applicable. A prior study found that TBS is a vertebral fracture predictor in subjects with adrenal incidentalomas [23]. A lower TBS value was found in those with prevalent fractures versus fracture-free controls [23], while in MACS versus overt Cushing’s syndrome, TBS was higher [24]. A previous study suggested that the cortisol/dehydroepiandrosterone-sulfate (DHEA-S) ratio might better correlate with TBS, but these are limited data [25]. Although normal values of TBS were traditionally considered at >1.350, recent data showed that the risk of fracture is highest in patients with a TBS of <1.23, intermediate in TBS between >1.23 and <1.31, and lowest at TBS > 1.31 [1,55]. Also, noting that people with diabetes and prediabetes have lower TBS values than healthy controls [56], this might not be strictly applicable in the diabetic population with the co-presence of mild cortisol anomalies due to an adrenal tumor. With the high prevalence of diabetes in the researched population, this aspect should also be accounted for.

Further research should include a more diverse population, including individuals with a higher fracture risk, and a longitudinal design to analyze the TBS- and irisin-based predictions. Whether only a subgroup of patients with MACS-positive and MACS-negative tumors is prone to TBS anomalies and changes in irisin levels (regardless of type 2 diabetes and age) is yet to be established. Steroid metabolomics (including adrenal androgens and even aldosterone in Connshing syndrome) might be connected to the microarchitecture damage and even exerkines interplay, more than shown by the traditional cortisol/ACTH testing.

Overall, the current data offer an exploratory perspective rather than a practical point for clinicians, but they might represent the first step in this complex landscape. The type 2 diabetic population and/or osteoporotic population harboring these tumors are treated based on the traditional recommendations for the general population with these ailments, regardless of the presence of the adrenal tumor.

## 5. Conclusions

In this pilot study, glycated hemoglobin A1c and HOMA-IR suggested a population with a certain level of glucose control, which might not be reflected in a severely damaged bone microarchitecture. The low TBS group had an increased age and menopause duration versus the group with normal TBS in patients without adrenal tumors, but not in the group with adrenal incidentalomas, which highlights a more complex influence on TBS values in these subjects. In group AI, patients with low TBS had lower osteocalcin, P1NP, and CrossLaps than those with normal TBS, thus, a decreased bone turnover. Irisin may be one of the additional factors in these tumors to reflect the hormonal burden. While irisin negatively correlated with osteocalcin, P1NP, and CrossLaps in tumor-free controls, this association was not found in the tumor group. Yet, in this group, a higher irisin was associated with a higher second-day cortisol after 1 mg DST and a lower baseline ACTH, pointing out a potential connection between the circulating miokine level and the adrenal hormonal profile.

To our best awareness, this is the first synchronous analysis of TBS and irisin levels in adrenal incidentalomas to address the bone status in relation to the glucose and adrenal status. Noting this is an exploratory analysis, further research will reveal the value of TBS and irisin testing for practitioners in the field of adrenal tumors, including multi-layered models/algorithms of the bone status prediction.

## Figures and Tables

**Table 1:** Demographics of the entire cohort, group AI, and group C.

Parameter	Entire Group (*N* = 81, 100%)	Group AI (*N* = 27, 33.33%)	Group C (*N* = 54, 66.67%)	*p*-Value	Normal
Age (years), mean ± SD	63.26 ± 8.82	65.30 ± 9.06	62.24 ± 8.59	0.142	
Years since menopause, mean ± SD	15.86 ± 9.50	17.26 ± 9.41	15.13 ± 9.56	0.349	
BMI (kg/sqm), mean ± SD	30.69 ± 5.76	30.18 ± 6.31	30.94 ± 5.50	0.577	<24.9
Glycated hemoglobin (%), mean ± SD	5.78 ± 0.44	5.78 ± 0.29	5.93 ± 0.28	0.940	4.8–5.9
Fasting glycaemia (mg/dL), mean ± SD	103.23 ± 12.91	102.91 ± 13.44	103.39 ± 12.78	0.880	80–115
Glycaemia—60 min in OGTT (mg/dL), mean ± SD	179.43 ± 43.79	184.66 ± 41.32	176.75 ± 45.27	0.506	
Glycaemia—120 min in OGTT (mg/dL), mean ± SD	131.50 ± 38.05	139.14 ± 45.31	127.68 ± 33.81	0.263	
Fasting insulin (µUI/mL), median (IQR)	6.68 (4.60, 12.35)	7.38 (4.67, 12.38)	6.65 (4.45, 12.69)	0.794	1.9–23
Insulin—60 min in OGTT (µUI/mL), median (IQR)	62.67 (40.50, 100.64)	56.45 (36.12, 93.37)	64.95 (42.48, 107.41)	0.593	
Insulin—120 min in OGTT (µUI/mL), median (IQR)	42.94 (20.54, 82.85)	42.93 (22.95, 76.73)	42.94 (18.78, 102.62)	0.853	
HOMA-IR, median (IQR)	1.45 (0.81, 3.02)	1.54 (0.80, 2.96)	1.42 (0.82, 3.19)	0.948	0.7–2
Type 2 diabetes, *N* (%)	55 (67.90)	18 (66.67)	37 (68.52)	0.865	

Abbreviations: AI = adrenal incidentaloma; C = control; BMI = body mass index; HOMA-IR = homeostasis model assessment of insulin resistance; IQR = interquartile range; OGTT = oral glucose tolerance test; SD = standard deviation.

**Table 2:** Hormonal, adrenal, and imaging profile in group AI.

Parameter	Group AI (*N* = 27, 33.33%)	Group C (*N* = 54, 66.67%)	*p*-Value	Normal Range
Baseline ACTH (pg/mL), mean ± SD	11.47 ± 4.59	15.12 ± 9.53	0.121	3–66
Baseline morning plasma cortisol (µg/dL), mean ± SD	11.11 ± 3.88	9.43 ± 3.75	0.176	6.2–19.4
Second day plasma cortisol after 1 mg DST (µg/dL), median (IQR)	1.59 (1.01, 2.03)	9.43 ± 3.75	0.260	<1.8
Largest tumor diameter (mm), mean ± SD	23.59 ± 11.90	NA		
Prevalence of the tumors with the second day plasma cortisol after 1 mg DST ≥ 1.8 µg/dL (MACS), *N* (%)	7 (25.97%)	NA		

Abbreviations: AI = adrenal incidentaloma; ACTH = adrenocorticotropic hormone; DST = dexamethasone suppression test; IQR = interquartile range; NA = not applicable; SD = standard deviation.

**Table 3:** Irisin, mineral metabolism assays, and bone turnover markers of the entire cohort, group AI, and group C.

Parameter	Entire Group (*N* = 81, 100%)	Group AI (*N* = 27, 33.33%)	Group C (*N* = 54, 66.67%)	*p*-Value	Normal Range
Circulating irisin (ng/mL), median (IQR)	71.17 (26.52, 146.46)	71.17 (20.16, 167.86)	77.64 (34.92, 133.28)	0.841	
Total serum calcium (mg/dL), mean ± SD	9.40 ± 0.49	9.45 ± 0.42	9.37 ± 0.52	0.491	8.4–10.2
Ionized serum calcium (mg/dL), mean ± SD	4.00 ± 0.29	4.00 ± 0.36	4.00 ± 0.26	0.999	3.9–4.9
Serum phosphorus (mg/dL), mean ± SD	3.66 ± 0.51	3.67 ± 0.48	3.65 ± 0.54	0.864	2.3–4.7
Serum creatinine (mg/dL), mean ± SD	0.76 ± 0.15	0.78 ± 0.13	0.75 ± 0.16	0.342	0.7–1.2
Serum urea (mg/dL), mean ± SD	38.04 ± 9.95	36.04 ± 8.02	39.04 ± 10.72	0.203	22–43
25-hydroxyvitamin D (ng/mL), mean ± SD	29.81 ± 8.02	30.93 ± 8.64	29.25 ± 7.71	0.380	30–100
Parathormone (pg/mL), mean ± SD	41.46 ± 12.58	42.96 ± 12.79	40.74 ± 12.54	0.464	15–65
Alkaline phosphatase (U/L), mean ± SD	77.14 ± 29.97	74.15 ± 30.00	78.63 ± 30.13	0.530	35–104
Osteocalcin (ng/mL), mean ± SD	22.04 ± 8.14	22.20 ± 8.16	21.96 ± 8.21	0.903	15–46
P1NP (ng/mL), mean ± SD	58.83 ± 24.86	55.81 ± 20.73	60.35 ± 26.75	0.451	20.25–76.31
CrossLaps (ng/mL), mean ± SD	0.39 ± 0.18	0.41 ± 0.17	0.39 ± 0.18	0.660	0.33–0.782
Lumbar BMD (g/sqcm), mean ± SD	1.074 ± 0.170	1.082 ± 0.165	1.069 ± 0.174	0.765	
Lumbar T-score (SD), mean ± SD	−0.83 ± 1.35	−0.78 ± 1.21	−0.86 ± 1.43	0.807	>−1
Femoral neck BMD (g/sqcm), mean ± SD	0.877 ± 0.123	0.878 ± 0.115	0.876 ± 0.128	0.941	
Femoral neck T-score (SD), mean ± SD	−0.89 ± 0.98	−0.92 ± 0.89	−0.88 ± 1.04	0.869	>−1
Total hip BMD (g/sqcm), mean ± SD	0.958 ± 0.134	0.954 ± 0.133	0.960 ± 0.136	0.859	
Total hip T-score (SD), mean ± SD	−0.37 ± 1.12	−0.40 ± 1.11	−0.34 ± 1.14	0.826	>−1
TBS, mean ± SD	1.355 ± 0.093	1.342 ± 0.088	1.362 ± 0.096	0.404	>1.350
Osteoporosis, *N* (%)	10 (12.35)	2 (7.41)	8 (14.81)	0.318	
Osteopenia, *N* (%)	37 (45.68)	13 (48.15)	24 (44.44)	0.999	
Normal DXA, *N* (%)	34 (41.98)	12 (44.44)	22 (40.74)	0.464	

Abbreviations: AI = adrenal incidentaloma; C = control; IQR = interquartile range; P1NP = procollagen 1 N-terminal propeptide; SD = standard deviation.

**Table 4:** BMD-DXA and TBS assessment of the entire cohort, group AI versus group C.

Parameter	Entire Group (*N* = 81, 100%)	Group AI (*N* = 27, 33.33%)	Group C (*N* = 54, 66.67%)	*p*-Value	Normal Range
Lumbar BMD (g/sqcm), mean ± SD	1.074 ± 0.170	1.082 ± 0.165	1.069 ± 0.174	0.765	
Lumbar T-score (SD), mean ± SD	−0.83 ± 1.35	−0.78 ± 1.21	−0.86 ± 1.43	0.807	>−1
Femoral neck BMD (g/sqcm), mean ± SD	0.877 ± 0.123	0.878 ± 0.115	0.876 ± 0.128	0.941	
Femoral neck T-score (SD), mean ± SD	−0.89 ± 0.98	−0.92 ± 0.89	−0.88 ± 1.04	0.869	>−1
Total hip BMD (g/sqcm), mean ± SD	0.958 ± 0.134	0.954 ± 0.133	0.960 ± 0.136	0.859	
Total hip T-score (SD), mean ± SD	−0.37 ± 1.12	−0.40 ± 1.11	−0.34 ± 1.14	0.826	>−1
TBS, mean ± SD	1.355 ± 0.093	1.342 ± 0.088	1.362 ± 0.096	0.404	>1.350
Osteoporosis, *N* (%)	10 (12.35)	2 (7.41)	8 (14.81)	0.318	
Osteopenia, *N* (%)	37 (45.68)	13 (48.15)	24 (44.44)	0.999	
Normal DXA, *N* (%)	34 (41.98)	12 (44.44)	22 (40.74)	0.464	

Abbreviations: AI = adrenal incidentaloma; C = control; BMD = bone mineral density; DXA = Dual-Energy X-Ray Absorptiometry; SD = standard deviation; TBS = trabecular bone score.

**Table 5:** Correlations between irisin, TBS, blood mineral metabolism assays, bone turnover markers, BMD, and adrenal (hormonal and CT) parameters within group AI and group C.

	Group AI (*N* = 27, 33.33%)		Group C (*N* = 54, 66.67%)	
Parameter	Irisin	TBS	Irisin	TBS
Total serum calcium (mg/dL)	r = −0.120 *p* = 0.551	r = 0.084 *p* = 0.578	r = −0.017 *p* = 0.858	r = −0.196 *p* = 0.074
Ionized serum calcium (mg/dL)	r = −0.008 *p* = 0.980	r = −0.067 *p* = 0.788	r = 0.012 *p* = 0.925	r = −0.268 *p* = 0.091
Serum phosphorus (mg/dL)	r = −0.372 *p* = 0.061	r = 0.169 *p* = 0.280	r = −0.110 *p* = 0.247	r = −0.087 *p* = 0.430
25-hydroxyvitamin D (ng/mL)	r = −151 *p* = 0.243	r = −0.052 *p* = 0.731	r = 0.083 *p* = 0.375	r = −0.061 *p* = 0.574
Parathormone (pg/mL)	r = 0.163 *p* = 0.243	r = −0.091 *p* = 0.554	r = 0.051 *p* = 0.586	r = −0.090 *p* = 0.406
Alkaline phosphatase (IU/L)	r = −0.151 *p* = 0.452	r = −0.123 *p* = 0.413	r = −0.044 *p* = 0.638	r = −0.054 *p* = 0.621
Osteocalcin (ng/mL)	r = −0.094 *p* = 0.648	r = 0.238 *p* = 0.121	**r = −0.252** *p * **= 0.007**	r = 0.023 *p* = 0.831
P1NP (ng/mL)	r = −0.068 *p* = 0.741	r = 0.221 *p* = 0.150	**r = −0.187 ** *p * **= 0.049**	r = −0.026 *p* = 0.816
CrossLaps (ng/mL)	r = 0.004 *p* = 0.986	**r = 0.364 ** *p * **= 0.018**	**r = −0.209 ** *p * **= 0.026**	r = −0.021 *p* = 0.848
Lumbar BMD (g/sqcm)	r = 0.119 *p* = 0.553	**r = 0.451 ** *p * **= 0.003**	r = −0.032 *p* = 0.743	r = 0.060 *p* = 0.582
Lumbar T-score (SD)	r = 0.110 *p* = 0.427	**r = 0.420 ** *p * **= 0.005**	r = −0.081 *p* = 0.413	r = 0.096 *p* = 0.380
Femoral neck BMD (g/sqcm)	r = 0.225 *p* = 0.259	r = 0.273 *p* = 0.068	r = −0.028 *p* = 0.776	r = 0.211 *p* = 0.053
Femoral neck T-score (SD)	r = 0.333 *p* = 0.090	r = 0.292 *p* = 0.053	r = −0.031 *p* = 0.755	r = 0.206 *p* = 0.060
Total hip BMD (g/sqcm)	r = 0.342 *p* = 0.080	r = 0.293 *p* = 0.051	r = 0.058 *p* = 0.563	r = 0.185 *p* = 0.093
Total hip T-score (SD)	r = 0.343 *p* = 0.080	r = 0.279 *p* = 0.067	r = 0.061 *p* = 0.545	r = 0.184 *p* = 0.098
TBS	r = 0.182 *p* = 0.405		r = −0.088 *p* = 0.419	
Baseline ACTH (pg/mL)	**r = −0.438 ** *p * **= 0.041**	r = −0.053 *p* = 0.753		
Baseline morning plasma cortisol (µg/dL)	r = −0.177 *p* = 0.430	r = 0.053 *p* = 0.753		
Second day plasma cortisol after 1 mg DST (µg/dL)	**r = 0.716 ** *p * **< 0.001**	r = 0.244 *p* = 0.174		
Largest tumor diameter (mm)	r = 0.110 *p* = 0.584	r = −0.266 *p* = 0.080		

Abbreviations: AI = adrenal incidentaloma; ACTH = adrenocorticotropic hormone; BMD = bone mineral density; C = control; DST = dexamethasone; TBS = trabecular bone score. The bold font means statistical significance.

**Table 6:** Multivariate linear regression model for predicting TBS (*N* = 81).

	TBS		
Parameter	B ± SE	β	*p*-Value
Constant	1.420 ± 0.113		**<0.001**
Age (years)	−0.004 ± 0.001	−0.358	**0.003**
Lumbar BMD (g/sqcm)	0.172 ± 0.070	0.293	**0.017**
Type 2 diabetes mellitus	0.011 ± 0.023	0.058	0.632
Adrenal incidentaloma	−0.022 ± 0.012	−0.112	0.329
Model summary	R Square = 0.236		**0.003**

Abbreviations: B = unstandardized regression coefficient; BMD = bone mineral density; SE = standard error; β = standardized regression coefficient; R square = coefficient of determination; TBS = trabecular bone score. The bold font means statistical significance.

**Table 7:** Distribution of patients in five-year age groups within the entire cohort, group AI, and group C.

Age Group (Years)	Entire Cohort	Group AI	Group C	*p*-Value
50–54	11 (13.58)	2 (7.41)	9 (16.67)	0.460
55–59	23 (28.40)	7 (25.93)	16 (29.63)	
60–64	12 (14.81)	4 (14.81)	8 (14.81)	
65–59	16 (19.75)	7 (25.93)	9 (16.67)	
70–74	7 (8.64)	1 (3.70)	6 (11.11)	
75–79	12 (14.81)	6 (22.22)	6 (11.11)	

Abbreviations: AI = adrenal incidentaloma, C = control.

**Table 8:** Irisin and TBS evaluation by five-year age groups.

Age Group (Years)	Irisin	*p*-Value	TBS	*p*-Value
50–54	25.60 (11.94, 113.60)	0.102	1.404 ± 0.069	0.118
55–59	91.85 (42.03, 186.35)		1.393 ± 0.100	
60–64	104.98 (42.58, 121.56)		1.348 ± 0.077	
65–59	40.90 (15.38, 100.22)		1.322 ± 0.085	
70–74	27.43 (16.52, 234.89)		1.314 ± 0.063	
75–79	152.80 (51.28, 193.98)		1.324 ± 0.117	

Abbreviation: TBS = trabecular bone score.

**Table 9:** Distribution of patients in body mass index groups within the entire cohort, group AI, and group C.

BMI Group	Entire Cohort	Group AI	Group C	*p*-Value
Normal	11 (13.58)	4 (14.81)	7 (12.96)	0.707
Overweight	27 (33.33)	10 (37.04)	17 (31.48)	
Grade I obesity	28 (34.57)	7 (25.93)	21 (38.89)	
Grade II obesity	8 (9.88)	4 (14.81)	4 (7.41)	
Grade III obesity	7 (8.64)	2 (7.41)	5 (9.26)	

Abbreviations: AI = adrenal incidentaloma; BMI = body mass index; C = control.

**Table 10:** Irisin and TBS evaluation by body mass index groups.

BMI Group	Irisin (ng/mL)	*p*-Value	TBS	*p*-Value
Normal	38.43 (9.34, 85.49)	**0.022**	1.366 ± 0.082	0.675
Overweight	54.75 (25.53, 120.88)		1.337 ± 0.091	
Grade I obesity	82.03 (26.06, 153.92)		1.360 ± 0.109	
Grade II obesity	156.66 (38.05, 261.21)		1.385 ± 0.062	
Grade III obesity	167.86 (121.79, 201.31)		1.346 ± 0.098	

Abbreviations: BMI = body mass index; TBS = trabecular bone score. Bold means statistical significance.

**Table 11:** Correlation of irisin and TBS with age groups and body mass index groups.

Parameter	Age Groups	BMI Groups
Irisin	r = 0.066 *p* = 0.421	**r = 0.259** *p * **= 0.002**
TBS	**r = −0.273 ** *p * **= 0.003**	r = 0.048 *p* = 0.615

Abbreviations: BMI = body mass index; TBS = trabecular bone score. Bold means statistical significance.

**Table 12:** The exploration of demographic characteristics, glucose, and musculoskeletal profile according to the study protocol on subjects with low (LT) versus normal TBS (NT) within study groups (AI and C).

	Group AI (*N* = 27)			Group C (*N* = 54)		
Parameter	Group LT (*N* = 13, 48.15%)	Group NT (*N* = 14, 51.85%)	*p*-Value	Group LT (*N* = 21, 38.89%)	Group NT (*N* = 33, 61.11%)	*p*-Value
Age (years), mean ± SD	65.46 ± 8.59	61.50 ± 7.79	0.267	66.95 ± 7.78	59.25 ± 7.53	**0.003**
Years since menopause, mean ± SD	18.62 ± 9.78	12.80 ± 7.57	0.135	19.00 ± 10.05	12.45 ± 8.33	**0.031**
BMI (kg/sqm), mean ± SD	30.43 ± 6.33	32.35 ± 4.86	0.435	30.75 ± 5.28	31.00 ± 5.79	0.887
Glycated hemoglobin (%), mean ± SD	5.62 ± 0.34	5.97 ± 0.48	0.051	5.69 ± 0.36	5.79 ± 0.58	0.499
Fasting glycaemia (mg/dL), mean ± SD	102.60 ± 15.68	103.61 ± 11.96	0.874	100.07 ± 12.20	104.21 ± 14.00	0.345
Glycaemia—60 min in OGTT (mg/dL), mean ± SD	168.18 ± 35.32	191.63 ± 47.06	0.244	166.13 ± 41.77	178.91 ± 52.02	0.470
Glycaemia—120 min in OGTT (mg/dL), mean ± SD	146.24 ± 52.59	119.60 ± 33.30	0.208	117.79 ± 37.87	124.15 ± 23.89	0.593
Fasting insulin (µUI/mL), median (IQR)	8.35 (5.52, 13.38)	9.16 (4.08, 15.35)	0.999	6.47 (4.23, 13.01)	5.86 (3.89, 9.89)	0.557
Fasting insulin—60 min in OGTT (µUI/mL), median (IQR)	59.35 (34.88, 106.96)	62.20 (30.43, 155.13)	0.897	65.02 (35.24, 113.38)	47.06 (36.98, 86.21)	0.581
Fasting insulin—120 min in OGTT (µUI/mL), median (IQR)	35.11 (19.17, 77.15)	49.06 (28.82, 65.43)	0.515	29.31 (8.73, 76.79)	38.91 (18.35, 55.75)	0.432
HOMA-IR, median (IQR)	1.77 (0.83, 2.90)	2.27 (0.91, 3.59)	0.602	1.27 (0.49, 2.39)	1.35 (0.77, 2.23)	0.813
Type 2 diabetes, *N* (%)	8 (61.54)	6 (42.86)	0.941	12 (57.14)	13 (39.39)	0.606
Circulating irisin (ng/mL), median (IQR)	38.43 (15.17, 142.59)	94.47 (19.69, 212.67)	0.483	101.27 (34.68, 185.27)	66.21 (44.05, 118.43)	0.639
Total serum calcium (mg/dL), mean ± SD	9.42 ± 0.35	9.46 ± 0.44	0.808	9.33 ± 0.54	9.33 ± 0.56	0.942
Ionized serum calcium (mg/dL), mean ± SD	3.98 ± 0.26	3.70 ± 0.18	0.195	3.98 ± 0.30	3.96 ± 0.24	0.838
Serum phosphorus (mg/dL), mean ± SD	3.62 ± 0.43	3.65 ± 0.53	0.878	3.70 ± 0.60	3.65 ± 0.53	0.765
25-hydroxyvitamin D (ng/mL), mean ± SD	32.07 ± 7.03	27.12 ± 6.61	0.101	29.29 ± 6.97	30.05 ± 7.19	0.731
Parathormone (pg/mL), mean ± SD	46.40 ± 13.24	38.23 ± 11.32	0.147	43.64 ± 14.39	37.85 ± 9.97	0.144
Alkaline phosphatase (U/L), mean ± SD	70.27 ± 23.97	77.20 ± 41.07	0.617	86.40 ± 41.18	73.84 ± 13.87	0.203
Osteocalcin (ng/mL), mean ± SD	18.51 ± 4.74	26.27 ± 10.78	**0.032**	22.08 ± 10.12	21.76 ± 7.93	0.911
P1NP (ng/mL), mean ± SD	44.68 ± 15.21	67.55 ± 22.86	**0.010**	62.17 ± 34.28	55.45 ± 21.18	0.466
CrossLaps (ng/mL), mean ± SD	0.31 ± 0.10	0.51 ± 0.17	**0.002**	0.40 ± 0.25	0.36 ± 0.13	0.516
Lumbar BMD (g/sqcm), mean ± SD	1.022 ± 0.121	1.208 ± 0.123	**0.002**	1.078 ± 0.166	1.095 ± 0.166	0.735
Lumbar T-score (SD), mean ± SD	−1.29 ± 0.97	0.10 ± 0.99	**0.003**	−0.97 ± 1.41	−0.76 ± 1.38	0.622
Femoral neck BMD (g/sqcm), mean ± SD	0.843 ± 0.111	0.922 ± 0.100	0.095	0.851 ± 0.128	0.922 ± 0.123	0.078
Femoral neck T-score (SD), mean ± SD	−1.15 ± 0.94	−0.49 ± 0.83	0.096	−1.10 ± 1.06	−0.49 ± 1.03	0.069
Total hip BMD (g/sqcm), mean ± SD	0.933 ± 0.144	1.013 ± 0.123	0.175	0.934 ± 0.157	0.993 ± 0.132	0.210
Total hip T-score (SD), mean ± SD	−0.56 ± 1.19	0.10 ± 1.04	0.179	−0.55 ± 1.29	−0.04 ± 1.10	0.192
TBS, mean ± SD	1.279 ± 0.052	1.423 ± 0.049	**<0.001**	1.291 ± 0.048	1.437 ± 0.074	**<0.001**
Osteoporosis, *N* (%)	2 (15.38)	0 (0.00)	0.486	4 (19.05)	2 (6.06)	0.663
Osteopenia, *N* (%)	6 (46.15)	4 (28.57)	0.768	11 (52.38)	9 (27.27)	0.758
Normal DXA, *N* (%)	5 (38.46)	10 (71.43)	0.414	6 (28.57)	22 (66.67)	0.341

Abbreviations: AI = adrenal incidentaloma; BMI = body mass index; BMD = bone mineral density; C = control; DXA = Dual-Energy X-Ray Absorptiometry; HOMA-IR = homeostasis model assessment of insulin resistance; LT = low trabecular bone score; IQR = interquartile range; NT = normal trabecular bone score; OGTT = oral glucose tolerance test; P1NP = procollagen 1 N-terminal propeptide; SD = standard deviation; TBS = trabecular bone score. Bold means statistical significance.

**Table 13:** Hormonal adrenal panel of patients with low TBS (LT) and normal TBS (NT) within group AI.

Parameter	Group LT (*N* = 13, 48.15%)	Group NT (*N* = 14, 51.85%)	*p*-Value
Baseline ACTH (pg/mL), mean ± SD	12.09 ± 4.34	10.86 ± 5.05	0.574
Baseline morning plasma cortisol (µg/dL), mean ± SD	10.73 ± 4.72	11.32 ± 3.03	0.761
Second day plasma cortisol after 1 mg DST (µg/dL), median (IQR)	1.10 (0.89, 1.47)	1.87 (1.59, 3.50)	0.055
MACS, *N* (%)	3 (23.08)	4 (28.57)	0.650
Largest tumor diameter (mm), mean ± SD	25.69 ± 13.33	23.30 ± 11.99	0.661

Abbreviations: AI = adrenal incidentaloma, LT = low trabecular bone score, NT = normal trabecular bone score, ACTH = adrenocorticotropic hormone, DST = dexamethasone suppression test, IQR = interquartile range, SD = standard deviation.

## Data Availability

The original contributions presented in this study are included in the article. Further inquiries can be directed to the corresponding authors.

## Abbreviations

The following abbreviations are used in this manuscript:

group AI	group with adrenal incidentaloma
ACTH	adrenocorticotropic hormone
BMD	bone mineral density
BMI	body mass index
ECLIA	electrochemiluminescence immunoassay
group C	control group
DST	dexamethasone
DHEA-S	dehydroepiandrosterone-sulfate
DXA	Dual-Energy X-Ray Absorptiometry
HOMA-IR	homeostasis model assessment of insulin resistance
group LT	group with a low trabecular bone score
MACS	mild autonomous cortisol secretion
N	number of patients
group NT	group with a normal trabecular bone score
Q	quartile
SD	standard deviation
SE	standard errors
TBS	trabecular bone score
